# Genomic Characterizations of Porcine Epidemic Diarrhea Viruses (PEDV) in Diarrheic Piglets and Clinically Healthy Adult Pigs from 2019 to 2022 in China

**DOI:** 10.3390/ani13091562

**Published:** 2023-05-06

**Authors:** Binghui Feng, Chen Li, Yuejia Qiu, Wenhao Qi, Ming Qiu, Jixiang Li, Hong Lin, Wanglong Zheng, Jianzhong Zhu, Nanhua Chen

**Affiliations:** 1 College of Veterinary Medicine, Yangzhou University, Yangzhou 225009, China; 2 Joint International Research Laboratory of Agriculture and Agri-Product Safety, Yangzhou 225009, China; 3 International Research Laboratory of Prevention and Control of Important Animal Infectious Diseases and Zoonotic Diseases of Jiangsu Higher Education Institutions, Yangzhou 225009, China; 4 Comparative Medicine Research Institute, Yangzhou University, Yangzhou 225009, China; 5 Jiangsu Co-Innovation Center for Prevention and Control of Important Animal Infectious Diseases and Zoonoses, Yangzhou University, Yangzhou 225009, China; 6 Key Laboratory of Animal Pathogen Infection and Immunology of Fujian Province, Fuzhou 350002, China

**Keywords:** porcine epidemic diarrhea virus, complete genome, recombination, diseased piglets, adult pigs

## Abstract

**Simple Summary:**

Diarrhea causes huge economic losses in the global swine industry. Porcine epidemic diarrhea virus (PEDV) has been identified as a direct causative pathogen of diarrheic diseases in piglets. PEDV can infect all ages of pigs; however, little is known about the prevalence and evolution of PEDV in adult pigs. Here, we explored the genomic characteristics of PEDV in both diarrheic piglets and clinically healthy adult pigs from 2019 to 2022 in China. We found that PEDV variants are not only prevalent in diseased piglets but are also circulating in adult pigs. In addition, intra- and inter-subgroup recombinants were detected in adult pigs, indicating that adult pigs may also serve as the host of viral reservoirs for rapid PEDV evolution. This study provides up-to-date information on PEDV’s prevalence and evolution in both diseased piglets and healthy adult pigs in China.

**Abstract:**

Porcine epidemic diarrhea virus (PEDV) is a major causative pathogen of diarrheic disease. In this study, the prevalence and evolution of PEDV was evaluated using intestinal samples collected from six provinces of China in 2019–2022. PEDV could not only be detected in diarrheic piglets but also in adult pigs without enteric diseases. The complete genomes of five temporal and geographical representative PEDV strains were determined. Genome-based phylogenetic analysis indicated that XJ1904-700 belongs to the G2-a subgroup, while the other strains are clustered within the S-INDEL subgroup. Recombination analyses supported that JSNJ2004-919 is an inter-subgroup recombinant from SD2014-like (G2-b), CHZ-2013-like (G2-b) and CV777-like (G1-b) isolates, while FJFZ2004-1017 is an intra-subgroup recombinant from XM1-2-like (S-INDEL) and LYG-2014-like (S-INDEL) isolates. Both JSNJ2004-919 and FJFZ2004-1017 were from adult pigs, providing evidence that adult pigs may also serve as the host of PEDV reservoirs for virus evolution. Overall, this study provides new insights into PEDV’s prevalence and evolution in both diseased piglets and clinically healthy adult pigs.

## 1. Introduction

Porcine epidemic diarrhea (PED) is a highly contagious enteric disease characterized by diarrhea, vomiting and dehydration [1]. PED was first identified in the United Kingdom and Belgium in the 1970s [2]. The etiologic agent, PED virus (PEDV), is a positive-sense single-stranded RNA virus belonging to the *Alphacoronavirus* genus of the *Coronaviridae* family within the *Nidovirales* order [3]. Before 2010, PEDV infection occurred sporadically or endemically [4]. In late 2010, PEDV variants first emerged in China and then were also found in the United States and other countries, resulting in huge economic losses in the global swine industry [5,6,7,8]. Based on the genetic diversity of the S gene, PEDV can be divided into group 1 (G1) and group 2 (G2). PEDV isolates before 2010 were grouped in G1, while the post-2010 PEDV variants (S-INDEL strains) were clustered within G2 [9]. Furthermore, G1 has evolved into two subgroups (G1-a and G1-b) and G2 has evolved into three subgroups (G2-a, G2-b and S-INDEL) [10].

The complete genome of PEDV is ~28 kb, containing at least seven open reading frames (ORFs) which encode the polyprotein (pp1ab), spike (S), ORF3 (hypothetical protein 3), envelope (E), membrane (M) and nucleocapsid (N) [11]. The S glycoprotein is on the surface of the viral particle, containing at least four neutralizing epitopes (COE, SS2, SS6 and 2C10) (Figure S1) [12,13,14]. The S protein includes S1 and S2 domains. The S1 interacts with host cellular receptors to mediate viral entry and the S2 mediates the fusion of viruses to host cells [10]. Substitutions, deletions and insertions occurring during virus evolution in the S protein could change the tissue tropism, pathogenicity and immunogenicity of PEDV [15,16,17,18]. A previous study confirmed that the S protein determines PEDV’s adaptability to monkey Vero cells and porcine LLC-PK1 cells [17]. Three amino acid mutations (A605E, E633Q and R891G) in the S protein of the Vero-cell-adapted PEDV strain DR13 were critical for its cell adaptation, which is probably achieved by altering the S2’ cleavage site and the RBD structure [15]. In addition, a 197 amino acid deletion in the N-terminal domain of S protein could attenuate a highly pathogenic PEDV strain (PC22A) in piglets, but the virus may lose important epitopes for inducing robust protective immunity [16]. Furthermore, an animal challenge study in gnotobiotic piglets indicated that the S1 subunit of the S protein is an important determinant of PEDV virulence [18].

Vaccination is a major strategy used for PED control. In China, several kill vaccines and three modified live vaccines (MLVs) (CV777-based, AJ1102-based and LW/L-based) have been widely used in Chinese swine herds. CV777 belongs to G1-b while AJ1102 and LW/L are clustered within the G2-a subgroup. However, new variants grouped in the G2-b and S-INDEL subgroups are currently prevalent in China [19]. In Japan, the PEDV 83P-5 (G1-a) strain was attenuated after 100 passages in Vero cells and employed as an intramuscular live-attenuated vaccine. In South Korea, two PEDV strains (SM98-1 and DR-13) were attenuated by serial passages and used as live or killed vaccines. In Philippines, the DR-13-based oral live vaccine was commercialized in 2011 [20]. In North America, the first PEDV vaccine (iPED) was developed by Harrisvaccines and licensed in 2013. The vaccine was developed with a truncated version of the PEDV S gene in the SirraVax^SM^ RNA Particle Technology platform. The second vaccine in the US was developed by Zoetis and became commercially available in 2014. The vaccine consists of an inactivated whole virus formulated with an adjuvant. The third vaccine was developed by the Vaccine and Infectious Disease Organization-InterVac in Canada. The vaccine is based on an inactivated virus formulated with an adjuvant [20]. Due to genetic differences between vaccine and field epidemic strains, commercial PEDV vaccines provide low to moderate protection against novel variants [6]. In addition, the wide usage of PEDV MLV also facilitates the occurrence of recombination events among PEDV MLVs and wild-type isolates [21].

Recombination is a critical evolutionary mechanism for RNA viruses [22]. PEDV may undergo recombination events that enable increased viral fitness. PEDV’s natural recombinants were frequently detected in previous studies. For instance, the XM1-2 strain is a recombinant from CHYJ130330 (G2-b) and HNQX-3 (S-INDEL) isolates [23]. CH/Liaoning25/2018 strain is derived from CN/GDZQ/2014 (G2-a) and SQ2014 (G1-b) strains [10]. The potential recombination events between G1-a and G2 strains led to the emergence of S-INDEL isolates, G1-b viruses and G2-c strains [21,24]. Although herd immunity and biosecurity are the most efficient strategies for preventing PED, the continuous emergence of new variants, including PEDV recombinants from vaccine strains and field isolates, has resulted in vaccination failure [21]. Moreover, the frequently occurring natural PEDV recombination events may also result in variations in virus pathogenicity [10,25].

PEDV infects all ages of pigs and may cause 100% mortality in neonatal piglets [26]. A majority of PEDV epidemiology studies have focused on PEDV infection in piglets. Very few studies have paid attention to PEDV infection in adult pigs. To investigate the up-to-date prevalence of PEDV in both piglets and adult pigs in China, an epidemiological investigation was performed using intestinal samples collected from both diarrheic piglets and adult pigs without enteric diseases from six provinces in 2019–2022 in this study. In addition, to investigate the evolution of PEDV in different ages of pigs, five representative PEDV genomes from both diarrheic piglets and adult pigs were determined and submitted to multiple alignment, phylogenetic analysis and recombination detection.

## 2. Materials and Methods

### 2.1. Clinical Sample Information

A total of 332 intestinal samples were collected from six provinces of China (Jiangsu, Fujian, Guangdong, Shandong, Henan and Xinjiang) from April 2019 to February 2022. A total of 58 of 332 samples were submitted from 13 farms located in seven cities of Jiangsu province. These samples were from diseased piglets with symptoms mainly including diarrhea, vomiting and death. In addition, 274 samples were randomly collected from 12 slaughter houses in 6 provinces (3 from Henan, 3 from Shandong, 2 from Jiangsu, 2 from Xinjiang, 1 from Fujian and 1 from Guangdong). These samples were from adult pigs without diarrheic symptoms. In this study, five representative PEDV positive samples were used for further evaluation.

### 2.2. Real-Time PCR Detection and Genomic Sequencing

Total RNAs were extracted from intestinal samples collected from dead piglets or slaughtered adult pigs using TRIpure Reagent (Aidlab, Beijing, China) according to the manufacturer’s instructions. cDNAs were generated by reverse transcription using HiScript III 1st Strand cDNA Synthesis Kit (+gDNA wiper, Vazyme, Nanjing, China). The obtained sample cDNA was detected by our previously described real-time RT-PCR assay for PEDV [11] using Q711-00 hamQ Universal SYBR qPCR Master Mix (Vazyme, Nanjing, China) and StepOne Plus Real-time PCR System (Thermo Fisher Scientific, Waltham, MA, USA). All PEDV real-time RT-PCR positive amplicons were confirmed by Sanger sequencing (Genewiz, Suzhou, China). Five PEDV-positive samples from five cities of four provinces (Kashi city of Xinjiang, Zhumadian city of Henan, Fuzhou city of Fujian, Nanjing and Nantong cities of Jiangsu) in 2019–2022 were selected as temporal and geographical representative samples and submitted to complete genome sequencing [23].

### 2.3. Sequence Alignment, Recombination and Phylogenetic Analyses

Multiple sequence alignment was performed using ClustalX 2.0 (Conway Institute, University College Dublin, Dublin, UK) based on the obtained five complete S proteins and five S proteins from representative PEDV isolates (CV777, AJ1102, AH2012, XM1-2 and XJ1904-34 strains). Phylogenetic tree based on 90 PEDV complete genomes was constructed using MEGA 6.06 (Pennsylvania State University, State College, PA, USA) with the neighbor-joining method [27]. The robustness of the phylogenetic tree was assessed by bootstrapping with 1000 replicates. In addition, recombination detection program 4 (RDP4) was used to detect potential recombination events [28]. Seven methods (RDP, GENECONV, BootScan, Maxchi, Chimaera, SiScan and 3Seq) embedded in RDP4 were used to detect cross-over events and breakpoints. The default settings were used and the highest acceptable *p* value cut-off was set at 0.01 as previously reported [29]. The detected recombination events were further evaluated by SimPlot 3.5.1 (Johns Hopkins University, Baltimore, MD, USA) [30].

## 3. Results

### 3.1. Chinese PEDV Strains from 2019 to 2022 from Different Ages of Pigs Obtained New Genetic Characteristics

**Figure 1:**
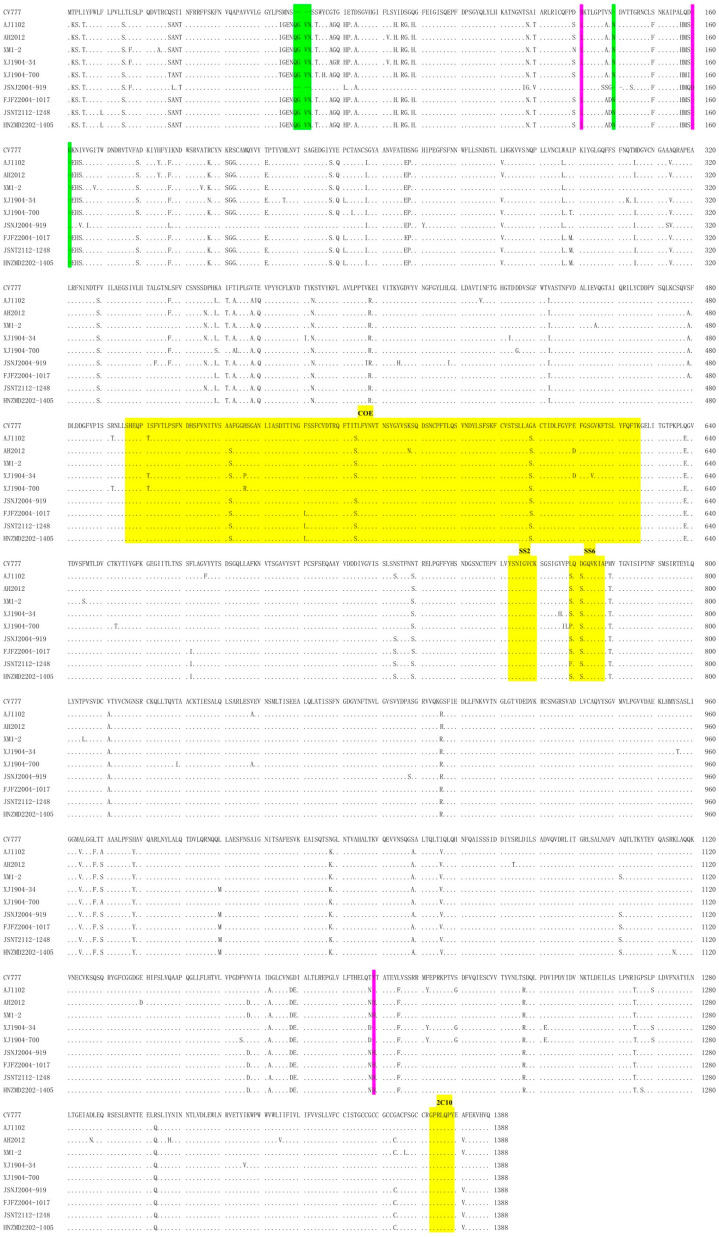
Multiple sequence alignment of PEDV S proteins. Four strains (XJ1904-700, FJFZ2004-1017, JSNT2112-1248 and HNZMD2202-1405) contain the unique insertions and deletion of G2 strains, while the JSNJ2004-919 strain is similar to G1 CV777 strain. The G2-variant-specific insertions (“QGVN” and “N”) and deletion (“G”) are highlighted in green. In addition, the additional deletions (at positions 131 and 1199) in XJ1904-700 strain and insertion (at position 160) in JSNJ2004-919 strain are marked in pink. The neutralizing epitopes are shown in yellow.

The real-time RT-PCR result showed that the overall PEDV-positive rate was 14.76% in our samples. PEDV could be detected in every year from 2019 to 2022 with positivity rates of 3.39–50.91%. In addition, PEDV could be detected in five provinces (except for Guangdong province) with positivity rates from 5.71% to 71.79%. Furthermore, a higher PEDV-positive rate was detected more in piglets that were within 10 days old (50.00%) than in adult pigs (7.30%) (Table 1). To evaluate the genetic characteristics and evolutionary properties of PEDV in Chinese swine herds, five PEDV-positive samples from five cities of four provinces (Kashi city in Xinjiang, Zhumadian city in Henan, Fuzhou city in Fujian and Nanjing and Nantong cities in Jiangsu) in 2019–2022 were selected as temporal and geographical representative samples and submitted to complete genome sequencing [23] (Table 2). The obtained genomes were submitted to GenBank with accession numbers of OQ269588-OQ269592. S protein alignment showed that four out of five PEDV strains (XJ1904-700, FJFZ2004-1017, JSNT2112-1248 and HNZMD2202-1405) have the same insertions (“QGVN” and “N”) and deletion (“G”) as other G2 variants, while the JSNJ2004-919 strain did not have these unique insertions and deletions, similar to the G1-b-attenuated CV777 strain (KT323979) (Figure 1). In addition, several substitutions were observed in the COE- and SS6-neutralizing epitopes of our PEDV strains. Moreover, new deletions (at positions 131 and 1199) were noticed in the XJ1904-700 strain and a new insertion (at position 160) was found in the JSNJ2004-919 strain.

### 3.2. Genomic Characterization Identified Novel PEDV Recombinants

**Figure 2:**
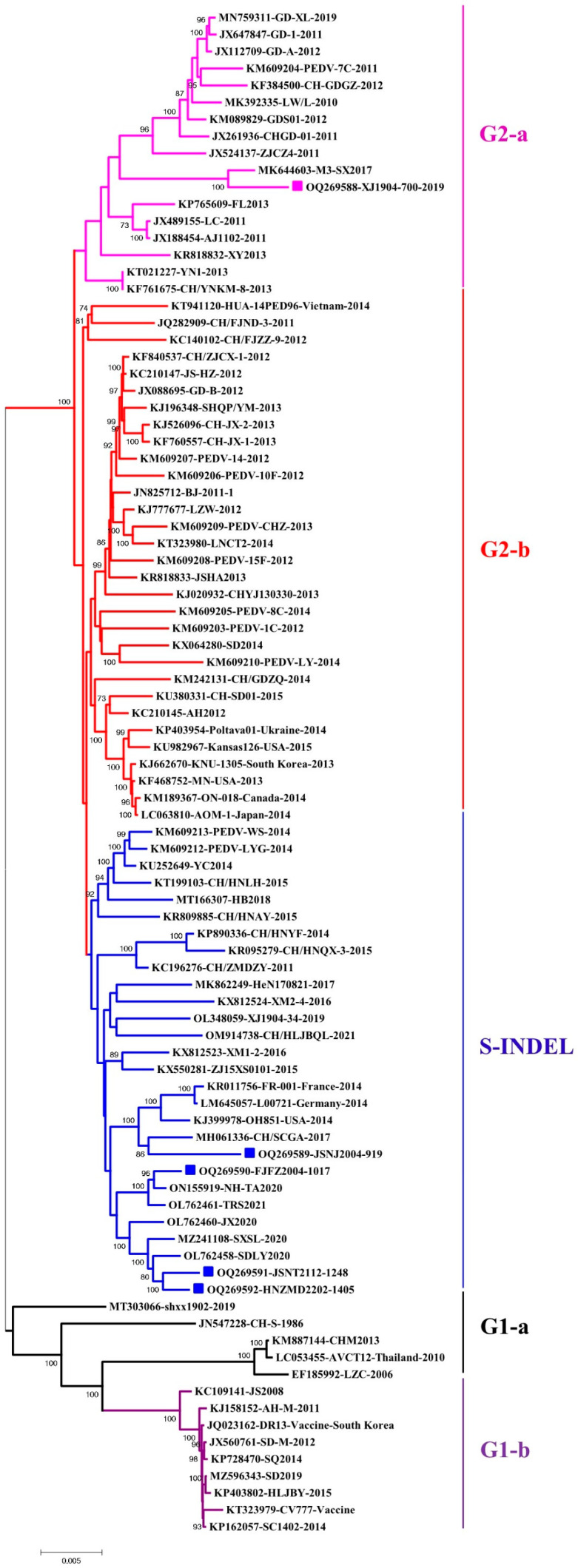
Genome-based phylogenetic analyses of PEDV strains. PEDV strains can be divided into G1 (G1-a and G1-b) and G2 (G2-a, G2-b and S-INDEL). The XJ1904-700 strain is clustered within G2-a subgroup, while the other four strains (JSNJ2004-919, FJFZ2004-1017, JSNT2112-1248 and HNZMD2202-1405) belong to the S-INDEL subgroup. Each subgroup is shown in distinct colors (G2-a in pink, G2-b in red, S-INDEL in blue, G1-a in black and G1-b in purple). The five PEDV strains identified in this study are highlighted with color squares.

**Figure 3:**
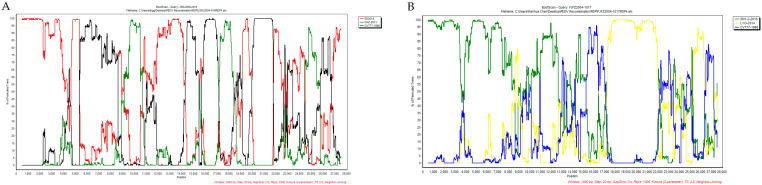
The cross-over regions in JSNJ2004-919 and FJFZ2004-1017 genomes. The cross-over regions are detected by SimPlot 3.5.1, which are basically consistent with the results from RDP4 analysis. (**A**) JSNJ2004-919 (S-INDEL) was derived from SD2014-like (G2-b), CHZ-2013-like (G2-b) and CV777-like (G1-a) strains. (**B**) FJFZ2004-1017 (S-INDEL) was recombined from XM1-2-like (S-INDEL) and LYG-2014-like (S-INDEL) isolates. The *y* axis shows the percentage of permutated trees employing a sliding window of 1000 bp and a step size of 20 bp. The other options including Kimura (2-parameter) distance model, 2.0 Ts/Tv ratio, neighbor-joining tree model and 1000 bootstrap replicates are used.

The complete-genome-based phylogenetic tree showed that the XJ1904-700 strain belongs to the G2-a subgroup, while the other four strains are clustered within the S-INDEL subgroup of G2 (Figure 2). Comparison results of the complete genome and each protein also supported that our five PEDV strains have higher homologies to G2 strains than to the G1 strain (Table 3). Noticeably, even though the S protein of the JSNJ2004-919 strain has higher similarity to the G1-b-attenuated CV777 strain (Figure 1 and Table 2), they did not group together in the genome-based phylogenetic tree. The recombination detection showed that JSNJ2004-919 (S-INDEL) was derived from inter-subgroup cross-over events by SD2014-like (G2-b), CHZ-2013-like (G2-b) and CV777-like (G1-a) strains, while FJFZ2004-1017 (S-INDEL) was generated by intra-subgroup cross-over events from XM1-2-like (S-INDEL) and LYG-2014-like (S-INDEL) isolates (Table 4). These results were further confirmed by SimPlot analysis (Figure 3).

## 4. Discussion

Diarrhea causes huge economic losses in the global swine industry and PEDV has been confirmed as a direct causative virus for diarrhea in piglets [7]. PEDV is one of the most rapidly evolving RNA viruses. Mutation and recombination frequently occur during the infection of PEDV in all ages of pigs [31]. Even though there are several commercial PEDV vaccines, they provide limited cross protection against novel variants [32]. Therefore, real-time monitoring, high-level biosafety and rational feeding management are critical for the prevention and control of PEDV-associated diseases. In this study, we identified that PEDV variants have been circulating in both diseased piglets and adult pigs without enteric diseases in distinct areas of China in the last five years. In addition, novel substitutions, deletions and insertions could be detected in 2019–2022 PEDV strains, indicating that Chinese PEDV isolates keep evolving and have obtained new genetic characteristics in the last five years. However, no unique genomic difference was noticed between PEDV strains from diarrheic piglets and healthy adult pigs. Remarkably, intra-subgroup and inter-subgroup recombination events were detected in PEDV strains from adult pigs, supporting that PEDV cross-over events could have occurred in adult pigs to generate novel recombinants.

Most PEDV epidemiological investigations are executed using samples collected from piglets. For instance, a previous study based on 435 samples collected from suckling piglets in Yunnan province during 2012–2017 showed a positivity rate of 17.47% [33]. Very few studies have addressed PEDV infection in adult pigs. Only an epidemiological survey in 2004 showed that the PEDV infection rate in piglets was 46.4% (4302/9212) with a mortality rate of 6.16% (256/9212), while the incidence rate in sows was 19.5% (356/1878) with no death in Guangxi province of China [34]. Consistent with previous studies, this study based on 332 intestinal samples collected from six provinces of China in 2019–2022 showed an overall PEDV-positive rate of 14.76% with a higher positivity rate in <10-day-old piglets (50%) than in adult pigs (7.30%). Noticeably, the PEDV-positive rates gradually decreased from 2019 (50.91%) to 2022 (3.39%), which is likely associated with the African Swine Fever (ASF) outbreak in 2018 and dramatically increased biosafety and management levels in Chinese pig farms. It is noteworthy that the PEDV-positive rates determined in this study are not able to represent the up-to-date condition of PEDV prevalence in China. More clinical samples should be analyzed to evaluate the overall prevalence of PEDV as well as PEDV prevalence per year/province/age in China.

PEDV has also caused epidemics, even pandemics, in other countries. In the United States, an epidemiological investigation in Colombia between 2014 and 2016 using 536 samples showed that the positivity rates decreased from 41.6% (2014) to 35.7% (2015) and then to 23.3% (2016). Complete-genome sequencing determined 21 PEDV genomes and identified recombinants within Colombian strains and between Colombian and Asian PEDV isolates [35]. In Europe, 40 swine feces collected from several PED outbreaks in Germany and other European countries were used for next-generation sequencing and 38 complete genomes (32 from Germany PED cases, 4 from Austrian samples and 2 from Romanian PED cases) were obtained. Even though these strains were closely related, two major clusters were identified, suggesting a single or simultaneous introduction of PEDV into Germany and Central Europe in 2014 [36]. In Japan, the complete genomes of 38 PEDV strains from diarrheal samples collected from 18 prefectures between 2013 and 2014 were determined using next-generation sequencing. Sequence comparison and phylogenetic analysis showed that the 38 Japanese PEDV strains have a higher correlation with PEDV variants from the United States and Korea in 2013–2014 than previously reported Japanese PEDV strains in 2006, suggesting that the re-emergence of PED outbreaks in Japan was caused by the introduction of recent PEDV strains from overseas [37]. In Vietnam, three complete genomes of PEDV strains isolated from 3-day-old piglets suffering diarrhea were characterized. Genome-based phylogenetic analysis revealed that Vietnamese PEDV isolates are grouped with Chinese PEDV variants in 2011–2012 but are genetically distinct from US isolates and classical PEDV isolates, suggesting that these Vietnamese isolates have originated from the same ancestor as the Chinese PEDV isolates [38].

Distinct PEDV isolates have been circulating and evolving in Chinese swine herds in last two decades. A majority of the post-2010 PEDV variants in China are clustered within G2, especially in the S-INDEL subgroup. In addition, novel PEDV variants can change the pathogenicity, immunogenicity and cell tropism during virus evolution in the field condition [15,16,39]. In this study, we determined the complete genomes of five representative PEDV strains from both diseased piglets and adult pigs without enteric diseases, which showed that they are evolutionary distinct. Further analysis showed that recombination plays a critical role in the rapid evolution of PEDV in adult pigs. Two PEDV strains (JSNJ2004-919 and FJFZ2004-1017) from adult pigs were detected as recombinants from inter-subgroup and intra-subgroup cross-over events. The JSNJ2004-919 strain was derived from inter-subgroup recombination by two G2-b strains (SD2014-like and CHZ-2013-like isolates) and a G1-a strain (CV777-like virus), while FJFZ2004-1017 was generated via intra-subgroup recombination from two S-INDEL isolates (XM1-2-like and LYG-2014-like viruses). To the best of our knowledge, JSNJ2004-919 is the first PEDV recombinant derived from two G2-b strains and a G1-b strain. Moreover, PEDV MLVs are widely used in Chinese swine herds, which also facilitates intra-subgroup and inter-subgroup recombinants between wild-type isolates or between wild-type and MLV strains [10,19,23,24,25]. For examples, the highly pathogenic CN/Liaoning25/2018 isolate (G1-a) was recombined from a highly pathogenic CN/GDZQ/2014 (G2-a) isolate (provided the S gene) and a low-pathogenic DR13-vaccine-like SQ2014 (G1-b) isolate (provided the remaining genomic regions) [10]. The HNAY isolate that was generated by natural cross-over events between two Chinese PEDV variants (HNZZ47 and GDS28 strains) displayed higher pathogenicity than two other non-recombinant isolates (HNXX and HB viruses) [25]. This study identified novel intra-subgroup and inter-subgroup recombinants in adult pigs, providing epidemiological evidence that adult pigs could serve as the host of virus reservoirs for rapid PEDV evolution. Even though the pathogenicity of our PEDV strains was not determined in this study, these PEDV strains with distinct genetic characteristics would likely also have differences in pathogenicity or immunogenicity, which deserves further investigation.

There are several areas that need to be addressed in following studies. First, PEDV variants in both diseased piglets and adult pigs should be isolated. However, propagating wild-type PEDV in cultured cells is still challenging due to the lack of knowledge about the mechanism of PEDV cell adaptation [15]. A PEDV reverse genetic system could be used to analyze the key determinants of cell adaptation for our strains. Secondly, even though XJ1904-700 and JSNT2112-1248 were from diarrheic piglets while JSNJ2004-919, FJFZ2004-1017 and HNZMD2202-1405 were from healthy adult pigs, it is unreasonable to propose the pathogenicity of these strains only based on clinical manifestations. The pathogenicity of distinct PEDV variants should be determined via a pig challenge study following Koch’s postulates. Thirdly, the influence of intra- and inter-subgroup recombination on PEDV’s pathogenicity also needs further investigation. The infectious clones of these recombinants could be used to construct chimeric viruses containing cross-over regions from distinct PEDV isolates. Additionally, animal challenge studies should be executed to determine the influence of the cross-over regions on PEDV’s pathogenicity.

## 5. Conclusions

We present up-to-date information of PEDV’s prevalence and evolution in both diseased piglets and adult pigs without enteric diseases in China. This study provides new insights into PEDV recombination in adult pigs.

## Figures and Tables

**Table 1:** PEDV infection status in six provinces of China from 2019 to 2022.

Year/Location	Sample No.	PEDV Positive No. ^1^	PEDV-Positive Percentages
Year			
2019	55	28	50.91%
2020	150	16	10.67%
2021	9	1	11.11%
2022	118	4	3.39%
Total	332	49	14.76%
Location			
Jiangsu	70	4	5.71%
Xinjiang	39	28	71.79%
Guangdong	23	0	0%
Henan	84	5	5.95%
Shandong	71	9	12.68%
Fujian	45	3	6.67%
Total	332	49	14.76%
Age			
<10 days old	58	29	50.00%
Adult	274	20	7.30%
Total	332	49	14.76%

^1^ PEDV was detected by real-time PCR assay as previously developed [11].

**Table 2:** Background information for the representative PEDV-positive samples use for genome sequencing.

No.	Name	Region (City, Province)	Date	Sample	Age	Symptom
1	XJ1904-700	Kashi, Xinjiang	April 2019	Intestine	5 days old	Diarrhea, death
2	JSNJ2004-919	Nanjing, Jiangsu	April 2020	Intestine	Adult pig	No enteric disease
3	FJFZ2004-1017	Fuzhou, Fujian	April 2020	Intestine	Adult pig	No enteric diseases
4	JSNT2112-1248	Nantong, Jiangsu	December 2021	Intestine	2 days old	Diarrhea, death
5	HNZMD2202-1405	Zhumadian, Henan	February 2022	Intestine	Adult pig	No enteric diseases

**Table 3:** Genome and each gene comparison of our PEDV strains with three representative PEDV MLV strains.

Gene/Genome		5′UTR	ORF1ab		S		ORF3		E		M		N		3′UTR	Complete Genome
**Encoded Protein**		**-**	**pp1ab**		**Spike Protein**		**Hypothetical Protein 3**		**Envelope Protein**		**Membrane Protein**		**Nucleocapsid Protein**		**-**	**-**
		**nt ^1^**	**nt**	**aa ^2^**	**nt**	**aa**	**nt**	**aa**	**nt**	**aa**	**nt**	**aa**	**nt**	**aa**	**nt**	**nt**
Identity (%) to CV777	XJ1904-700	97.60 ^3^	97.08	97.54	92.96	92.14	-	-	96.10	96.05	97.94	97.79	96.83	97.73	97.90	96.30
	JSNJ2004-919	98.97	97.08	97.60	**94.82**	**94.14**	-	-	94.81	96.05	97.65	96.90	95.93	96.83	98.20	**96.55** ^4^
	FJFZ2004-1017	98.97	97.30	97.85	93.16	92.50	-	-	95.67	96.05	97.50	97.35	95.55	96.60	96.44	96.43
	JSNT2112-1248	98.29	97.15	97.77	93.30	92.43	-	-	95.24	96.05	97.06	96.90	95.93	96.83	98.20	96.36
	HNZMD2202-1405	98.29	97.21	97.94	93.32	92.36	-	-	94.81	94.74	97.21	96.90	95.85	96.60	98.50	96.41
Identity (%) to AJ1102	XJ1904-700	98.29	97.93	98.42	97.72	98.05	99.11	100	99.57	100	99.41	100	97.74	97.96	99.10	97.96
	JSNJ2004-919	99.66	98.55	98.75	**94.62**	**94.52**	96.15	97.32	98.27	100	98.53	98.23	95.93	97.05	98.80	**97.77**
	FJFZ2004-1017	99.66	98.82	98.94	97.26	98.20	96.15	97.32	99.13	100	98.38	98.67	96.15	96.83	97.03	98.35
	JSNT2112-1248	99.66	98.57	98.89	97.31	98.05	96.00	97.32	98.70	100	97.94	98.23	96.08	97.05	98.80	98.17
	HNZMD2202-1405	99.66	98.68	99.10	97.48	98.05	96.15	97.32	98.27	98.68	98.09	98.23	96.00	96.83	99.10	98.29
Identity (%) to LW/L	XJ1904-700	98.63	97.87	98.10	97.55	97.18	98.52	98.66	99.13	98.68	99.12	99.12	97.66	97.73	99.10	97.90
	JSNJ2004-919	100	97.84	98.08	**94.45**	**93.65**	95.56	95.98	97.84	98.68	98.24	97.35	95.85	96.83	98.80	**97.24**
	FJFZ2004-1017	100	98.14	98.32	96.95	96.97	95.56	95.98	98.70	98.68	98.09	97.79	96.08	96.60	97.03	97.81
	JSNT2112-1248	99.32	97.93	98.22	96.92	96.68	95.41	95.98	98.27	98.68	97.65	97.35	96.00	96.83	98.80	97.65
	HNZMD2202-1405	99.32	98.05	98.45	97.04	96.68	95.56	95.98	97.84	97.37	97.80	97.35	95.93	96.60	99.10	97.76

^1^ nt is the abbreviation of nucleotide. ^2^ aa is the abbreviation of amino acid. ^3^ The numbers indicate the percentage (%) of similarity between each comparison. For instance, the first number indicates 97.60% nucleotide similarity between XJ1904-700 and CV777 in 5′UTR. ^4^ The G2 JSNJ2004-919 strain shares the highest similarity to G1 CV777 MLV strain but the lowest to G2 AJ1102 and LW/L MLV strains when compared with the other four strains. The corresponding percentages are underlined and shown in bold.

**Table 4:** Summary of potential recombination events identified by the RDP v.4.71 (University of Cape Town, Cape Town, South Africa).

Recombinant Virus	Parental Virus		Breakpoint ^1^			Score for the Seven Detection Methods Embedded in RDP4						
	Major	Minor	Region	Begin	End	RDP	GENECONV	BootScan	MaxChi	Chimaera	SiScan	3Seq
JSNJ2004-919	SD2014 (KX064280)	CHZ-2013 (KM609209)	ORF1b	10,270	17,314	1.474 × 10^−19^	3.378 × 10^−12^	- ^2^	3.691 × 10^−7^	1.611 × 10^−7^	5.710 × 10^−11^	1.509 × 10^−13^
JSNJ2004-919	SD2014 (KX064280)	CV777 (KT323979)	ORF1b-S	15,053	21,317	-	-	-	3.612 × 10^−19^	3.788 × 10^−17^	5.225 × 10^−7^	8.881 × 10^−16^
FJFZ2004-1017	XM1-2 (KX812523)	LYG-2014 (KM609212)	ORF1b-S	17,651	21,558	8.796 × 10^−29^	2.824 × 10^−32^	2.835 × 10^−34^	9.863 × 10^−20^	4.821 × 10^−15^	2.963 × 10^−24^	4.440 × 10^−16^

^1^ The breakpoint position in the genome of the tested virus. ^2^ “-” indicates that the recombination event is not significant. The *p* value cut-off was set at 0.01.

## Data Availability

The obtained PEDV genomes in this study have been submitted to GenBank with accession numbers of OQ269588-OQ269592.

## Supplementary Materials

The following supporting information can be downloaded at https://www.mdpi.com/article/10.3390/ani13091562/s1, Figure S1. Schematic diagram of PEDV genome. PEDV genome includes a 5′ cap structure, 5′untranslating region (UTR), seven open reading frames (ORFs), 3′UTR and the 3′-poly-A tail. ORF1a and ORF1b encode polyprotein pp1ab, while the other ORFs encode structural proteins including spike (S), accessory protein ORF3, envelope (E), membrane (M) and nucleocapsid (N). Spike protein contains a signal peptide (SP), a sialic acid binding region (SIA), fusion peptide (FP), heptad repeat domains (HR1 and HR2), transmembrane domain (TM) and cytoplasmic domain (CP). All these regions are shown in gray. In addition, the Spike protein has four neutralizing epitopes, including CO-26K equivalent epitope (COE, 499–638 amino acids), SS2 (^748^YSNIGVCK^755^), SS6 (^764^LQDGQVKI^771^) and 2C10 (^1368^GPRLQPY^1374^). These neutralizing epitopes are marked in blue.
