# Supplementary material for: Genomic Characterizations of Porcine Epidemic Diarrhea Viruses (PEDV) in Diarrheic Piglets and Clinically Healthy Adult Pigs from 2019 to 2022 in China
Source: Animals (Basel). 2023 May 6;13(9):1562. doi: 10.3390/ani13091562 (PMC10177568; doi:10.3390/ani13091562)
Supplement: Supplementary file 1 [file animals-13-01562-s001.zip › animals-2296279-supplementary.pdf]

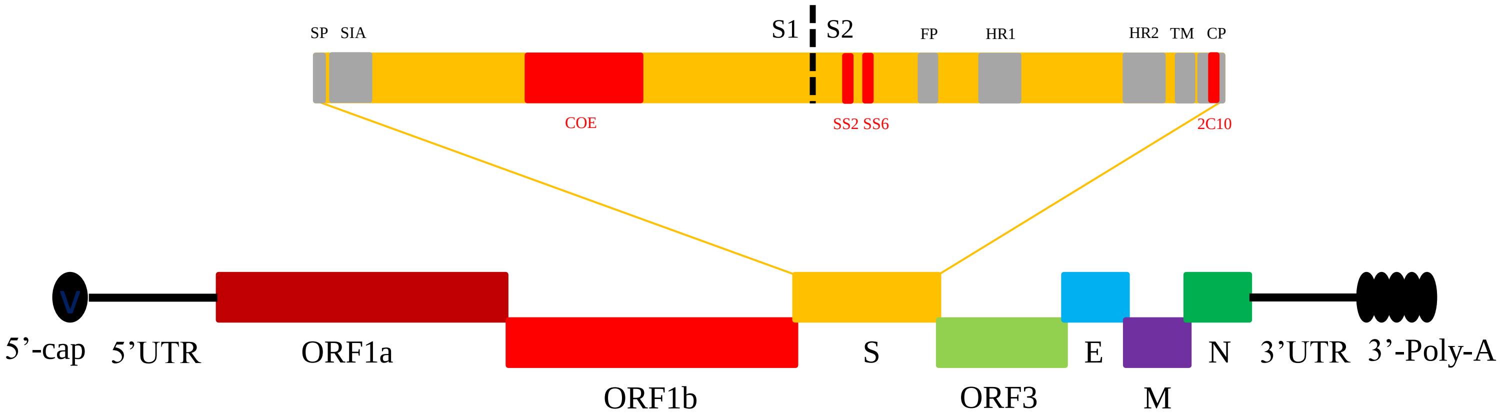

Figure S1. Schematic diagram of PEDV genome. PEDV genome includes a 5’ cap structure, 5’untranslating region (UTR), seven open reading frames (ORFs), 3’UTR and the 3’-poly-A tail. ORF1a and ORF1b encode polyprotein pp1ab, while the other ORFs encode structural proteins including spike (S), accessory protein ORF3, envelope (E), membrane (M) and nucleocapsid (N). Spike protein contains a signal peptide (SP), a sialic acid binding region (SIA), fusion peptide (FP), heptad repeat domains (HR1 and HR2), transmembrane domain (TM) and cytoplasmic domain (CP). All these regions are shown in gray. In addition, the Spike protein has four neutralizing epitopes, including CO-26K equivalent epitope (COE, 499-638 amino acids), SS2 (^{748}YSNIGVCK^{755}), SS6 (^{764}LQDGQVKI^{771}) and 2C10 (^{1368}GPRLQPY^{1374}).
